# Broad-Spectrum Antimicrobial Activity and Improved Stability of a D-Amino Acid Enantiomer of DMPC-10A, the Designed Derivative of Dermaseptin Truncates

**DOI:** 10.3390/antibiotics9090627

**Published:** 2020-09-21

**Authors:** Yu Zai, Yuan Ying, Zhuming Ye, Mei Zhou, Chengbang Ma, Zhanzhong Shi, Xiaoling Chen, Xinping Xi, Tianbao Chen, Lei Wang

**Affiliations:** 1 School of Pharmacy, Queen’s University Belfast, Belfast BT9 7BL, UK; yzai01@qub.ac.uk (Y.Z.); y.ying@qub.ac.uk (Y.Y.); zye04@qub.ac.uk (Z.Y.); m.zhou@qub.ac.uk (M.Z.); c.ma@qub.ac.uk (C.M.); x.chen@qub.ac.uk (X.C.); t.chen@qub.ac.uk (T.C.); l.wang@qub.ac.uk (L.W.); 2 Department of Natural Sciences, Faculty of Science and Technology, Middlesex University, London NW4 4BT, UK; z.shi@mdx.ac.uk

**Keywords:** antimicrobial peptide, D-amino acid, protease stability, *Galleria mellonella* larva model

## Abstract

DMPC-10A (ALWKKLLKK-Cha-NH_2_) is a 10-mer peptide derivative from the N-terminal domain of Dermaseptin-PC which has shown broad-spectrum antimicrobial activity as well as a considerable hemolytic effect. In order to reduce hemolytic activity and improve stability to endogenous enzymes, a D-amino acid enantiomer (DMPC-10B) was designed by substituting all L-Lys and L-Leu with their respective D-form amino acid residues, while the Ala^1^ and Trp^3^ remained unchanged. The D-amino acid enantiomer exhibited similar antimicrobial potency to the parent peptide but exerted lower cytotoxicity and hemolytic activity. Meanwhile, DMPC-10B exhibited remarkable resistance to hydrolysis by trypsin and chymotrypsin. In addition to these advantages, DMPC-10B exhibited an outstanding antibacterial effect against Methicillin-resistant *Staphylococcus aureus* (MRSA) and *Klebsiella pneumoniae* using the *Galleria mellonella* larva model and displayed synergistic activities with gentamicin against carbapenem-resistant *K. pneumoniae* strains. This indicates that DMPC-10B would be a promising alternative for treating antibiotic-resistant pathogens.

## 1. Introduction

Nowadays, infectious diseases are an increasingly serious phenomenon, mainly due to an increase in antibiotic-resistant pathogens. Ten million people worldwide are estimated to die from infections by drug-resistant bacteria by 2050 [1]. Antimicrobial peptides (AMPs) have received attention for their broad-spectrum antimicrobial activity and low toxicity against mammalian cells, and they are less likely to produce drug resistance [2,3,4,5] as they can target phospholipids and disrupt the cell membrane of pathogens. However, some disadvantages of naturally occurring peptides, such as poor chemical and physical stability, a tendency toward aggregation, and a short half-life and fast elimination, limit their potential for clinical applications [6].

Dermaseptins are a class of cationic AMPs that were discovered from the skin of *Phyllomedusa* frogs, usually consisting of 28–34 amino acids. They can form an amphipathic α-helix in amphiphilic environments, which makes them bind easily to bacterial lipid bilayers [7,8]. Dermaseptins contain a conserved Trp residue in position three and a unique internal motif, -AAA/GKAAL/G/NA-, and exert broad-spectrum antimicrobial activity against a range of ESKAPE pathogens [7,8]. It has been suggested that the N-terminal domain of dermaseptin demonstrates selectivity during the interaction with the bacterial cell membrane, while the C-terminal helix mainly exhibits nonspecific membrane lytic activity [9,10]. Previous research of N-terminal peptide fragments of dermaseptins has shown that 16- to 19-mer truncated peptide retains similar antimicrobial potency, while shorter sequences (less than 13 amino acid residues) lose massive antimicrobial activity [11,12]. In our previous study, we designed an optimized decapeptide derived from a dermaseptin N-terminal derivative, DMPC-10A (ALWKKLLKK-Cha-NH_2_), by balancing the net charge and hydrophobicity. Additionally, cyclohexanylalanine (Cha) was introduced at the C-terminus to improve the membrane permeabilization [13]. Although DMPC-10A has demonstrated broad-spectrum inhibitory activity, it still induces a considerable hemolytic effect [13].

D-amino acids are enantiomeric residues that are occasionally found in the constitutions of natural proteins. The peptide bond formed by D-amino acids is resistant to enzymatic hydrolysis. On the other hand, the substitution of D-enantiomer in AMPs has demonstrated a change in secondary structure that decreases the cytotoxicity to zwitterionic cell membranes and improves stability [14,15,16,17]. In previous studies, researchers have substituted all of the amino acids in naturally occurring membrane-active peptides such as cecropin A, magainin 2 amide, and melittin with D-type amino acids, and these enantiomers were demonstrated to be resistant to enzymatic degradation, showing activity similar to that of their natural form [18,19,20]. However, an enantiomer may not be suitable as a therapeutic agent because its extremely long half-life may increase the side effects. An alternative method by which to overcome this limitation is to design partial D-amino acid substitution analogues of natural peptides [15]. Some studies have pointed out that D-type leucine and lysine substitution can significantly improve the stability of peptides against trypsin degradation and have exhibited more efficient killing of Gram-negative and Gram-positive bacteria [16,21]. In this study, we designed an analogue of DMPC-10A with substitutions of L-Lys and L-Leu by respective enantiomeric D-amino acid residues and further assessed antimicrobial activity using the *Galleria mellonella* larva model.

## 2. Results

### 2.1. Structure Analysis of DMPC-10B

**Figure 1:**
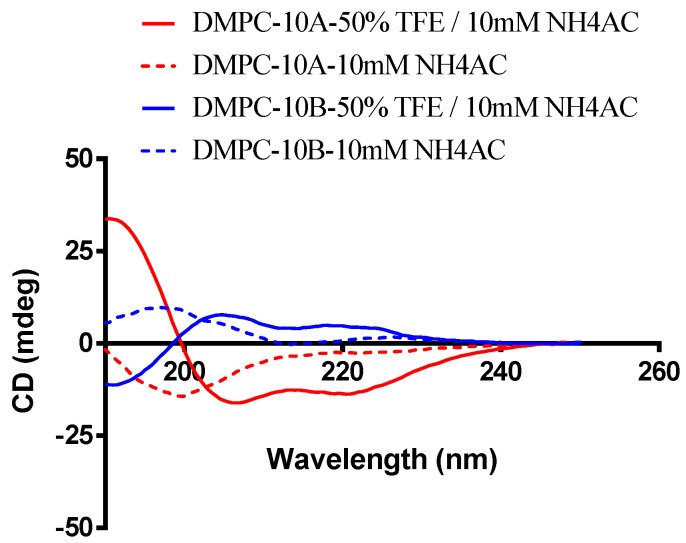
Secondary structure analysis of DMPC-10A and DMPC-10B; 50-μM peptides analyzed by treatment with 10 mM NH_4_AC and 50% trifluoroethanol (TFE)/10 mM NH_4_AC. The spectra were averaged over three consecutive scans and the solvent circular dichroism (CD) signal was subtracted.

**Figure 2:**
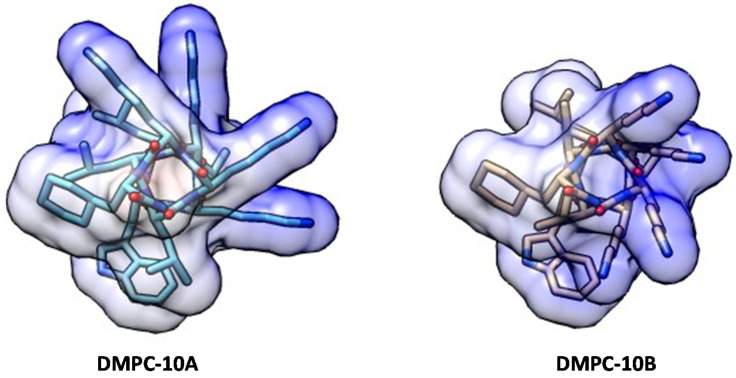
The conformation of DMPC-10A and DMPC-10B simulated by molecular modeling. Both peptides were represented as a helical structure. The coulombic surface of each peptide was applied to display the positively charged residues (blue area).

The epimerization of the D-amino acid close to the N-terminus has been determined to have an impact on the average helicity of a peptide, which is an important parameter for the antimicrobial activity and selectivity of AMPs [22]. Unlike DMPC-10A, which could form an obvious α-helix structure in an amphipathic environment, DMPC-10B has demonstrated a left-handed α-helix structure in trifluoroethanol (TFE) (Figure 1). Based on the helical secondary structures, molecular modeling was performed to show the conformation of both peptides (Figure 2).

### 2.2. Antimicrobial Activities

DMPC-10A and DMPC-10B were able to inhibit the growth of the bacteria strains in this study. The cell viability of each bacterium treated by the respective peptide is showed in Figure S1. DMPC-10B demonstrated broad-spectrum inhibitory activity, where its minimum inhibitory concentrations were around 2 to 64 μM (Table 1). Compared with DMPC-10A, the antibacterial performance of DMPC-10B for *E. coli* and methicillin-resistant *Staphylococcus aureus* (MRSA) was improved four-fold and two-fold, respectively, while for *K. pneumoniae* (ATCC 43816), the MIC showed a two-fold increase. Generally, two peptides exhibit similar antimicrobial potency.

Overall, the antimicrobial activity of DMPC-10A and DMPC-10B was proven to be affected by the presence of different cations (Figure S2). Cations such as Mg^2+^ and Ca^2+^ showed a similar influence on MICs at two concentrations (2 and 5 mM) (Table 2). However, compared with DMPC-10A, DMPC-10B is more sensitive to Na^+^ in the environment. Additionally, 10% of fetal bovine serum (FBS) has less influence on the antimicrobial activity of two peptides than the cations.

### 2.3. Enzyme Stability

**Figure 3:**
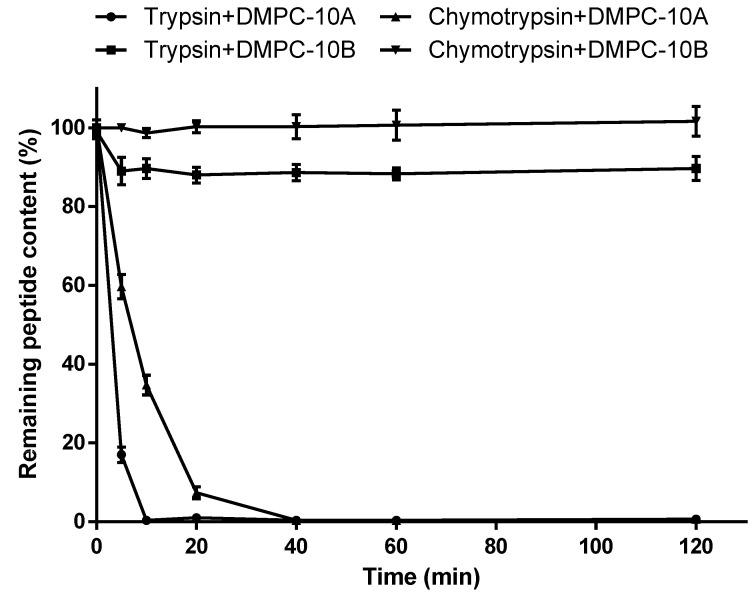
The amount of intact peptides of DMPC-10A and DMPC-10B undergoing in vitro hydrolysis by trypsin and chymotrypsin in 120 min. The error bar represents the standard deviation (SD) of three replicates.

Unlike the similar pattern of DMPC-10A and DMPC-10B in the medium containing cations or FBS, they demonstrated different degrees of tolerance towards the hydrolysis by trypsin and chymotrypsin (Figure 3). This revealed that trypsin completely cleaved DMPC-10A in 10 min with the appearance of fragment peaks in the HPLC chromatogram traces (Figure S3), whereas it could not degrade DMPC-10B completely within 2 h. Similarly, chymotrypsin cleaved DMPC-10A in 40 min, while DMPC-10B exhibited resistance to the hydrolysis. This proves that the substitution of D-amino acids could significantly improve the stability of DMPC-10B in the presence of endogenous enzymes.

### 2.4. Hemolytic and Cytotoxic Activity

**Figure 4:**
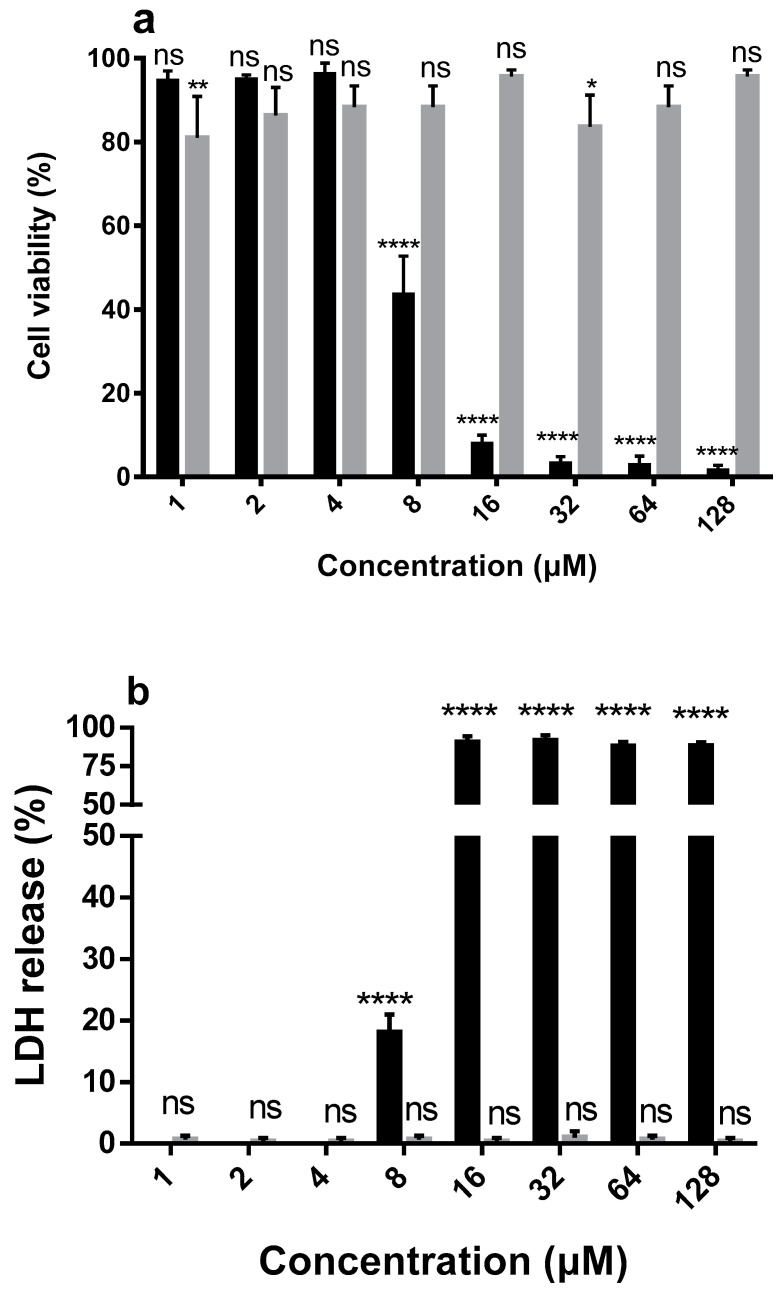

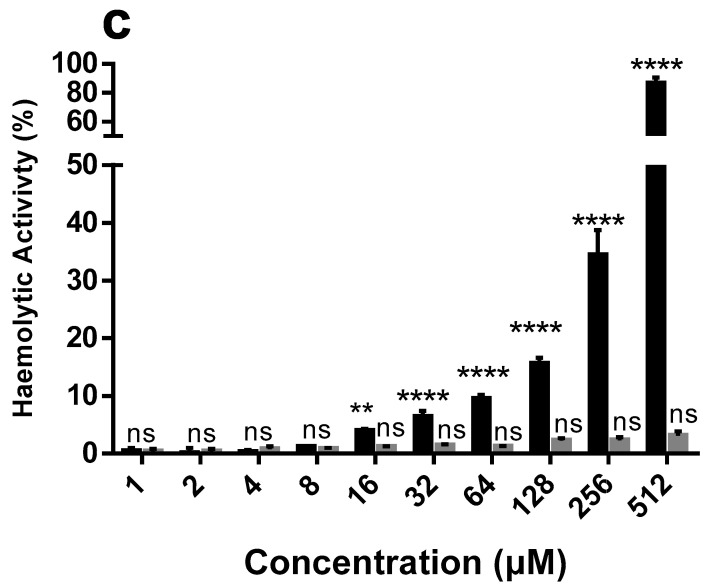
The cytotoxicity evaluation of DMPC-10A (black) and DMPC-10B (grey). (**a**) The cell viability of HaCat (human keratinocyte cell line) treated by DMPC-10A and DMPC-10B. (**b**) The release of lactate dehydrogenase (LDH) from HaCat cells in the presence of DMPC-10A and DMPC-10B. (**c**) The hemolytic activities of DMPC-10B and DMPC-10A at concentrations of 1 to 512 μM. The percentage was calculated based on the effect induced by a positive control, 1% Triton X-100. Treatment with phosphate-buffered saline (PBS) was used as a negative control. Error bars indicate standard deviation (SD) of 15 replicates in three tests (5 replicates each time). The statistical significance was calculated using one-way ANOVA and is indicated as ns (nonsignificant difference), * (*p* < 0.05), ** (*p* < 0.01), and **** (*p* < 0.0001).

We evaluated the cytotoxicity of DMPC-10A and DMPC-10B on the human keratinocyte cell line, HaCat, using 3-(4,5-dimethylthiazol-2-yl)-2,5-diphenyltetrazolium bromide (MTT) and lactate dehydrogenase (LDH) assays. DMPC-10B did not induce any cytotoxicity on HaCat at a concentration up to 128 µM (Figure 4). Similarly, DMPC-10B did not exhibit any hemolytic activity on the horse erythrocytes. However, compared with DMPC-10B, DMPC-10A induced more severe hemolysis and cytotoxicity.

### 2.5. Membrane Permeability

**Figure 5:**
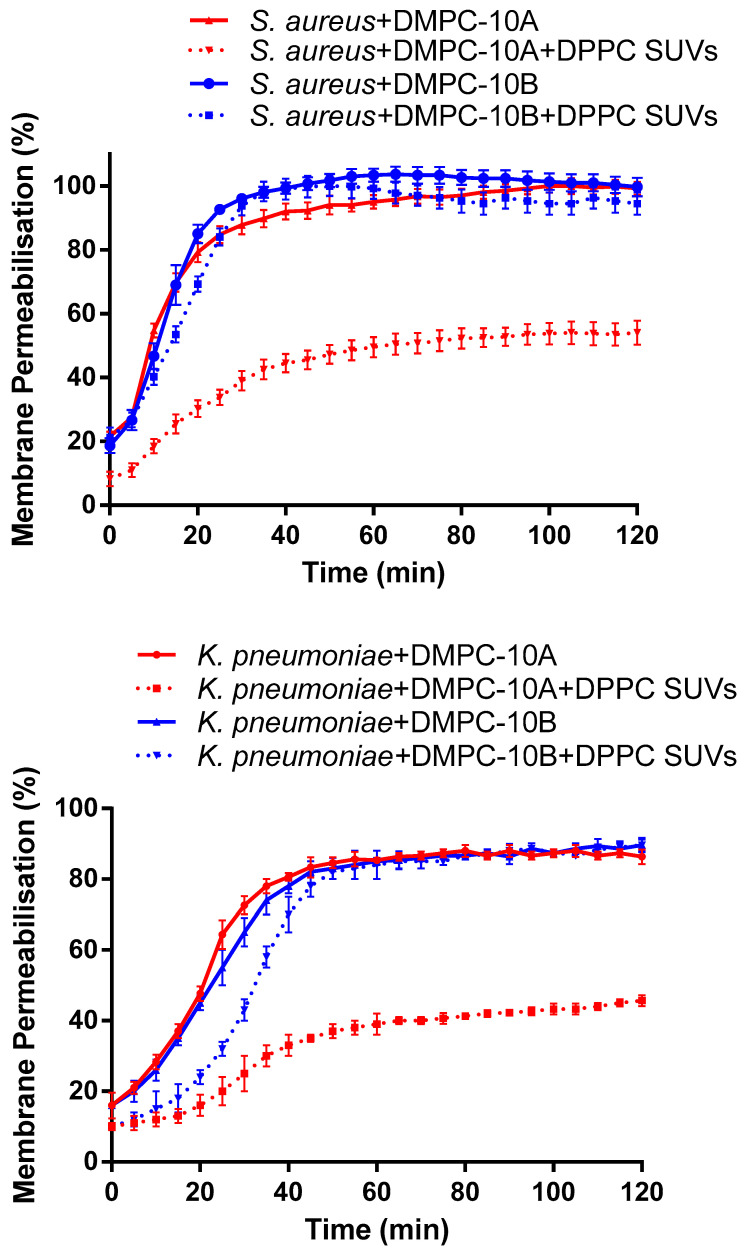
Kinetics of membrane permeabilization of DMPC-10A and DMPC-10B on *S. aureus* (NCTC 10788) and *K. pneumoniae* (ATCC 43816) at 16 µM. To determine the lipid selectivity of both peptides, 1 mg/mL of dipalmitoylphosphatidylcholine (DPPC) small unilamellar vesicle (SUVs) was added. The percentage of membrane permeabilization was measured using the bacterial cells treated with 70% isopropanol. The error bar represents the standard deviation (SD) of five replicates.

As the results show (Figure 5), both DMPC-10A and DMPC-10B permeabilize the cell membrane of *S. aureus* and *K. pneumoniae*. As we know, phosphatidylcholine (PC) is predominant in the outer leaflet of the plasma membrane of mammalian cells [23]. Considering that DMPC-10B exerted low cytotoxicity and hemolytic activity on mammalian cells, 1 mg/mL of dipalmitoylphosphatidylcholine (DPPC) small unilamellar vesicle (SUVs) was added to further explore the selectivity of the peptides to the lipid layer with different lipid constitutions. It showed that DPPC SUVs interfered with the interaction between DMPC-10A and the bacterial cell membrane, decreasing the membrane permeabilization effect on *S. aureus* and *K. pneumoniae*. However, the membrane permeabilization of DMPC-10B was not affected significantly by the presence of DPPC SUVs. Furthermore, the docking analysis of both peptides in the 1-palmitoyl-2-oleoyl-sn-glycero-3-phosphoethanolamine (POPE) and 1-palmitoyl-2-oleoyl-sn-glycero-3-phosphocholine (POPC) lipid bilayers showed that DMPC-10B revealed a slightly higher binding affinity to the POPE lipid bilayer (Figure S4).

### 2.6. Antibiofilm Activities

**Figure 6:**
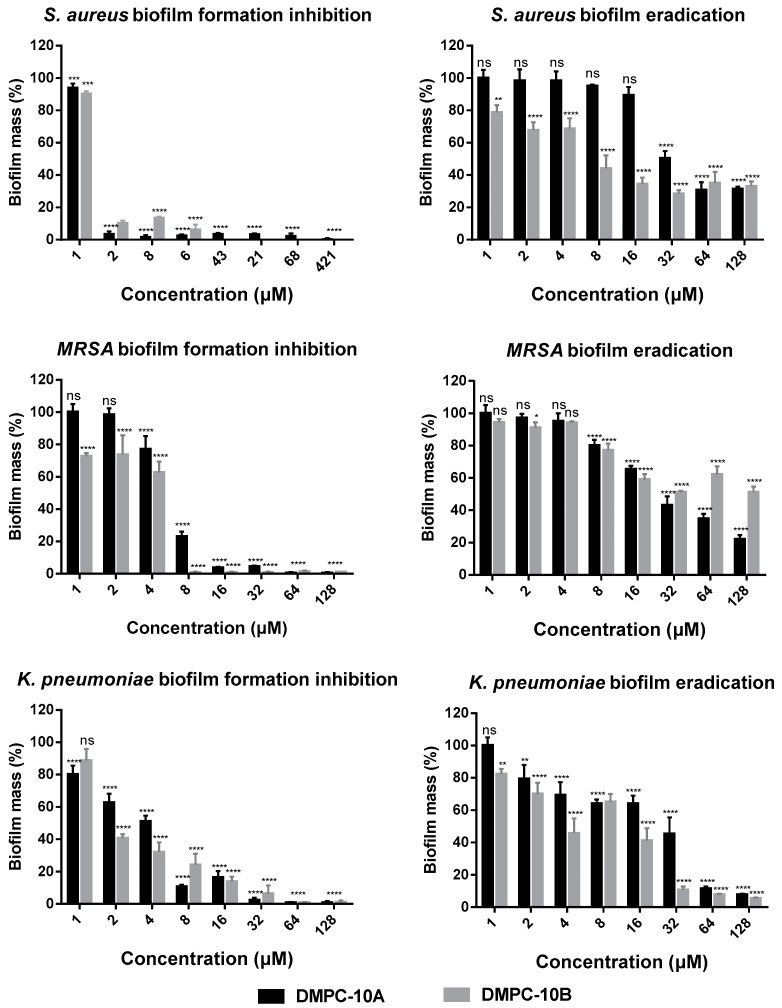
The percentage of biofilm mass of *S. aureus* (NCTC 10788), MRSA (NCTC 12493), and *K. pneumoniae* (ATCC 43816) after treatment with DMPC-10A and DMPC-10B in the biofilm inhibition and eradication assays. The error bar represents the standard deviation (SD) of 15 replicates in three tests (5 replicates each time). The statistical analysis was performed by one-way ANOVA and the statistical significance between the peptide concentrations and the negative control (medium treatment) is indicated as ns (nonsignificant difference), * (*p* < 0.05), ** (*p* < 0.01), *** (*p* < 0.001) and **** (*p* < 0.0001).

*S. aureus* (NCTC 10788), MRSA (NCTC 12493) and *K. pneumoniae* (ATCC 43816) were selected for studying the anti-biofilm activity of DMPC-10A and DMPC-10B, due to their low MICs against the respective strains. As shown in Figure 6, DMPC-10A and DMPC-10B effectively inhibited the formation of biofilms of *S. aureus* and MRSA, but the effect is less potent against *K. pneumoniae*. Especially, they exhibited a relatively stronger effect on *S. aureus*. Meanwhile, both peptides displayed around 50% biofilm eradication effect at the high concentrations ranging from 32 to 128 µM against *S. aureus* and MRSA, while they showed more potent effects against *K. pneumoniae* at 64 µM and 128 µM (Figure 6). Overall, DMPC-10B exerted a similar effect to DMPC-10A, except that it demonstrated slightly effective activity to eradicate the biofilm of MRSA.

### 2.7. Antimicrobial Synergy Study

Previous research has confirmed that many AMPs are able to lyse the biological membrane and generate pores by different mechanisms, such as toroidal pore and barrel stave models, which could consequently allow the antibiotics to bypass the bacterial cell membrane in large numbers and kill the bacteria [24,25], thereby achieving a synergistic effect. The principle is the same as the clinical application of the combination of the membrane-rupturing antibiotics and the intracellular-targeting antibiotics. Gentamicin can bind to 30S rRNA to inhibit the protein synthesis of bacteria [26,27]. The kinetics of uptake of gentamicin involve an initial energy-independent phase associated with ionic binding to the cell surface and cytoplasmic membrane. This is followed by two energy-dependent phases, a slow initial rate of uptake termed energy-dependent phase I (EDP-I) and a second accelerated rate termed energy-dependent phase II (EDP-II) [28]. Although gentamicin demonstrates poor cell permeability, it was reported that the combination of gentamicin and the highly cationic and amphipathic α-helical peptide PMAP-36 or PRW4 could enhance the antimicrobial effect and exert a synergistic effect, which may result from the easier access to the cytoplasmic membrane for gentamicin after the disruption of the outer membrane by the two peptides [29,30]. Therefore, we speculated that DMPC-10B, with the ability to rupture and penetrate the cell membrane, could help the uptake of gentamicin and achieve a synergistic effect.

*K. pneumoniae* (ATCC BAA 1705) and *K. pneumoniae* (ATCC BAA 2342), two kinds of KPC-producing *K. pneumoniae* strains, show resistance to cephamycins and carbapenems in addition to the substrate range of extended-spectrum β lactamases (ESBLs) [31,32]. Actually, a nosocomial outbreak caused by gentamicin-resistant *Klebsiella pneumoniae* occurred in the Neonatal Intensive Care Unit (NICU) [33]. Therefore, there is an urgent need for improving the therapeutic approaches against multidrug-resistant bacteria; to achieve this, the synergistic application of AMPs and conventional antibiotics would be a promising strategy.

The synergistic effects between DMPC-10B and gentamicin or norfloxacin were studied using a checkerboard assay (Figure S5). The combination of DMPC-10B and gentamicin shows a synergistic effect on *K. pneumoniae* (ATCC 43816), *K. pneumoniae* (ATCC BAA 1705), and *K. pneumoniae* (ATCC BAA 2342). The lowest fractional inhibitory concentration index (FICI), which is used to measure the combined effect of different compounds, was 0.375 for three bacteria (Table 3). In the meantime, DMPC-10B showed only an addictive effect in the combination with norfloxacin.

### 2.8. Treatment of Larvae Infected with MRSA and K. pneumoniae with DMPC-10B

**Figure 7:**
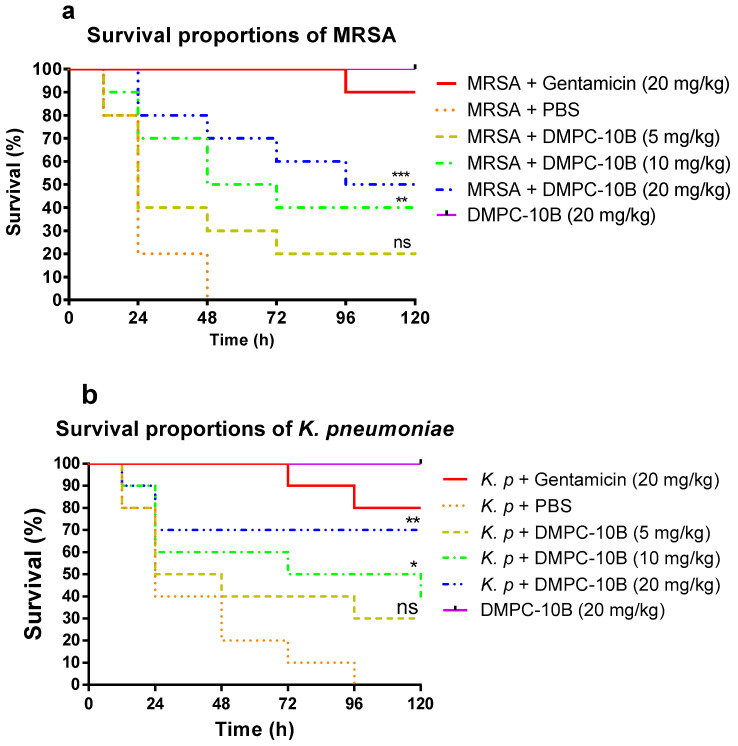
The mortality of *Galleria mellonella* larvae infected with (**a**) methicillin-resistant *Staphylococcus aureus* (MRSA) (NCTC 12493) and (**b**) *K. pneumoniae* (ATCC 43816). The infected larvae were treated with gentamicin (20 mg/kg), PBS, and different doses of DMPC-10B, respectively. The larvae without infection were treated with 20 mg/kg of DMPC-10B, which was applied to assess the potential toxicity of DMPC-10B to the hosts. The statistical analyses were performed by log-rank test to compare with the negative control (PBS treatment). The statistical significance is indicated as ns (nonsignificant difference), * (*p* < 0.05), ** (*p* < 0.01), and *** (*p* < 0.001).

The mortality of the larvae infected by MRSA and *K. pneumoniae* was significantly decreased by the treatment of DMPC-10B (Figure 7). In addition, the highest dose of DMPC-10B did not induce any death of healthy larvae. DMPC-10B significantly improved the mortality of infected larvae. It exhibited a stronger effect on larvae infected with *K. pneumoniae* at a higher concentration (20 mg/kg), with around 70% survival, while only 50% larvae infected by MRSA survived at the same dose of DMPC-10 in 5 days. In contrast, the effects of the lower doses (5 mg/kg and 10 mg/kg) are similar in treatment of the infections by MRSA and *K. pneumoniae*.

## 3. Discussion

AMPs, exerting remarkable antimicrobial effects, especially against multidrug-resistant bacterial strains, have emerged as promising alternatives to antibiotics [34,35]. DMPC-10A is a potent N-terminal derivate of dermaseptin that has demonstrated broad-spectrum antimicrobial activity. The introduction of a Cha residue at the C-terminus has enhanced the membrane permeabilization effect on the bacterial cell membrane. Meanwhile, due to the strong hydrophobicity of the cyclohexyl group of Cha residue, DMPC-10A also revealed considerable hemolytic activity. Additionally, that DMPC-10A consists of L-amino acid contributes to the obstacle of poor stability in the presence of proteases, which limits the clinical application [36]. As previous research pointed out, a feasible method to overcome these limitations is to substitute the L-amino acids at the most susceptible site with D-amino acids [15]. Obviously, D-amino acid substitution does not change the net positive charge of the original peptide, but the configuration and the action related to the recognition of chiral targets would be affected [16,37]. Most AMPs kill bacteria by the destruction of the cell membrane through pore-forming activity [38,39]. Additionally, it is well established that the all-D enantiomers (the natural peptides consist of all D-amino acids) with the left-handed α-helical structure are equally active and pore-forming [19,40]. Compared with the all-D enantiomers, the bioactivity of the analogues with partial substitution by D-amino acid is more unpredictable. For some cases, such as the peptide W3R6, partial substitution (D-Arg-W3R6) made it exert stronger antimicrobial activity than the all-D enantiomer (D-W3R6) [41]. However, for another AMP, polybia-CP, the partial D-lysine substitution derivative (D-Lys-CP) showed slightly weaker antimicrobial activity than the all all-D enantiomer (D-CP) [42]. Herein, we replaced the L-amino acids in the helical region of DMPC-10A with D-amino acids to produce a left-handed α-helical structure in the membrane-mimicking environment (Figure 1) to retain the amphipathic feature for the interaction with the lipid bilayer. Meanwhile, ^1^Ala and ^3^Trp still remained as L-form because we aimed to reduce some helicity of the peptide, which might decrease the interaction with the zwitterionic lipid that is commonly distributed in the mammalian cell membrane [8,13,43].

As the CD spectrum showed, DMPC-10B formed a left-handed α-helical structure, allowing it to interact with the cell membrane. However, the helicity of DMPC-10B is lower than that of DMPC-10A, which could be deduced from the reduced peak area at 208 and 222 nm. Additionally, the antimicrobial activity of DMPC-10B was not severely affected by the substitution of the D-amino acids. Meanwhile, the antimicrobial activity of DMPC-10B against *E. coli* was increased significantly. On the other hand, both peptides were influenced by the cations in the environment, where the antimicrobial potency of DMPC-10B was affected more severely than that of DMPC-10A. This may result from the competitive binding of cations and cationic peptide on the cell membrane of bacteria [44]. However, the larger helical segment of DMPC-10A could still facilitate binding with the lipid bilayer.

On the contrary, we found that phosphatidylcholine (PC) greatly affected the membrane permeabilization of DMPC-10A, but it did not extensively impact the effect of DMPC-10B. This indicates that DMPC-10B possesses selectivity towards the cell membrane with different lipid constitutions. It is also consistent with the explanation of the different activities of DMPC-10A and DMPC-10B towards the mammalian cell line and the erythrocytes. Again, we assume that DMPC-10A may exert two ways for binding to the lipid bilayer, one is the electrostatic interaction and another is the amphipathic interaction by the helical formation. Therefore, it could interact with the negatively charged bacterial cell membrane as well as the zwitterionic lipid bilayer. However, due to the less helical configuration of DMPC-10B, electrostatic interaction could be the predominant pattern in the process of killing mechanism. It was deduced that DMPC-10B was less effective on HaCat cells and red blood cells, as their outer leaflet of the plasma membrane contains predominant zwitterionic PC [23]. However, the main lipid components of the bacterial cell membrane are negatively charged phosphatidylethanolamine (PE) and phosphatidylglycerol (PG) [45]. Besides this, the previous study demonstrated that peptide substituted by D-amino acid could not deeply insert into the hydrophobic core of the zwitterionic lipid bilayer [17].

In addition, DMPC-10B showed great stability in trypsin and chymotrypsin environment. In nature, the majority of proteins and peptides consist of L-amino acids that can be hydrolyzed by endogenous enzymes. However, D-amino acid associated peptide bonds could alter the direction of the side chain and twist the backbone of the main chain, which prevents the binding to the enzyme [37]. Moreover, the L-Trp in DMPC-10B, which can be cleaved by chymotrypsin, was not cleaved within 2 h. It is speculated that Trp is connected with D-amino acids, where the spatial structure could be changed to place into the rection pocket of the enzyme. This situation is similar to that of the presence of Pro at P1′ position, which can block hydrolysis by trypsin or chymotrypsin [46].

Notably, the growth of tested antibiotic strains in our study was effectively inhibited by DMPC-10B individually as well as in combination with conventional antibiotics. As we know, AMPs are excellent antimicrobial candidates which have been considered to be less subjected to the development of resistance [47]. It has also been proven as a promising solution to amplify the potency of conventional antibiotics through drug combination. For instance, the antimicrobial potency of amoxicillin has been enormously enhanced in the combined use of clavulanic acid [48]. Herein, DMPC-10B displayed synergistic activities with gentamicin against *K. pneumoniae*. The mechanism of action of gentamicin involves creating fissures in the outer membrane of the bacterial cell and inhibition of bacterial protein synthesis by binding to 30S ribosomes [49,50]. The previous study showed that the combination of gentamicin and highly cationic and amphipathic α-helical peptides would exert a synergistic effect, as it could be the easier for gentamicin to traverse the cytoplasmic membrane after the permeabilization of the outer membrane by the peptides [29,30]. Although the mechanism of the synergetic effect between DMPC-10B and gentamicin remains unrevealed, DMPC-10B may possess direct and selective membrane permeabilizing activity to facilitate the translocation of gentamicin into the bacterial cells and initiate the process to bind with intracellular 30S ribosomes, which leads to the enhancement of the antimicrobial activity [51]. The application of the combination of AMPs and conventional antibiotics could be a prospective strategy to combat multidrug resistant pathogens and decrease the side effect of antibiotics at high doses [52].

Wax moth (*G. mellonella*) larva infection model was used to assess the efficacy of DMPC-10B against MRSA and *K. pneumoniae* in vivo. Compared with the other AMPs applied using the same model, such as Japonicin-2LF [53], DMPC-10B exerted relatively stronger efficacy. Japonicin-2LF prevented around 50% mortality at 50 mg/kg in treatment of MRSA infection, but DMPC-10B achieved similar efficacy at 20 mg/kg. Additionally, Japonicin-2LF exhibited over 80% hemolysis and induced considerable cytotoxicity [53]. The more potent antimicrobial efficacy of DMPC-10B might be related to the resistance to enzymatic degradation, which might prolong the half-life in vivo. Collectively, the design of DMPC-10B not only reduces the hemolysis and cytotoxicity but also contributes to the enhancement of the antimicrobial efficacy. Although DMPC-10B did not exhibit improved efficacy compared with gentamycin in vitro, it still exerted similar potency to gentamycin at the dose of 20 mg/kg against *K. pneumoniae*, suggesting that DMPC-10B has great potential for development as an antibiotic alternative to combat the rising issue of antibiotic resistance.

## 4. Materials and Methods

### 4.1. Solid Phase Peptide Synthesis

Fmoc-chemistry peptide synthesis was performed for all peptides in this study, using a Tribute Peptide Synthesiser (Protein Technologies, Tucson, AZ, USA), which was described in the previous study [53]. Briefly, 0.3 mmol of each Fmoc amino acid was weighted and mixed with an equal amount of 2-(1H-benzotriazol-1-yl)-1,1,3,3-tetramethyluronium hexafluorophosphate (hexafluorophosphate benzotriazole tetramethyl uronium, HBTU) in the loading vial. Rink amide resin (250 mg) was employed as the solid phase for the synthesis of peptide chain as well as providing the C-terminal amide for all peptides. In the synthesis process, the peptide bonds were coupled in the presence of HBTU that was dissolved by 1 M N-methylmorpholine (NMM) in dimethylformamide (DMF), followed by the deprotection of α-NH_2_ by 20% (*v*/*v*) piperidine in DMF. Once the synthesis was accomplished, a 25 mL cleavage cocktail (trifluoroacetic acid (TFA)/water/thioanisole/1,2-Ethanedithiol = 94/2/2/2 (*v*/*v*/*v*/*v*)) was added to the resin–peptide matrix for 2–4 h at room temperature for releasing the peptide chains from the resin as well as deblocking the side chains. The synthetic peptides were purified by reverse-phase HPLC and lyophilized for functional tests.

### 4.2. Molecular Modeling and Docking

Molecular modeling was employed using UCSF Chimera software package [54]. The two peptides were built, and the conformations were adjusted with the minimization simulation, using the non-solvation environment. The coulombic surface was presented to show the positive charge of the side chains. For molecular docking, AutoDock vina [55] was employed to simulate the interaction between the energy adjusted peptides and different lipid bilayers. The POPE and POPC lipid bilayer models were provided by Tieleman [56].

### 4.3. Circular Dichroism (CD)

The secondary structure of all synthetic peptides was investigated using a JASCO J-815 CD Spectropolarimeter (JASCO Inc., Tokyo, Japan) as described in the previous study [57]. Each peptide was prepared in 10 mM ammonium acetate (NH_4_AC; Sigma-Aldrich, Gillingham, UK) buffer (pH 7.4) and the membrane-mimic solution 50/50 (*v*/*v*) 2,2,2-trifluoroethanol (TFE; Sigma-Aldrich, Gillingham, UK)/10 mM NH_4_AC (pH 7.4), at a final concentration of 50 µM, respectively. The solution was loaded in a 1-mm thickness quartz cuvette and analyzed at room temperature. The range of the wavelength for analysis was programmed from 190 to 250 nm. The peptide sample was scanned by a 1 nm bandwidth light at 0.5 nm data pitch at the speed of 100 nm/min. The final spectrum of each sample was generated by averaging data from three scans.

### 4.4. Antimicrobial Assay

The antimicrobial activity of the peptides was generally assessed through the determination of the minimum inhibitory concentrations (MICs) and the minimum bactericidal concentration (MBCs) using the broth-dilution method, as described in the previous study [57]. Gram-positive bacteria *Staphylococcus aureus* (NCTC 10788) and *Enterococcus faecalis* (NCTC 12697) and Gram-negative bacteria *Escherichia coli* (NCTC 10418), *Pseudomonas aeruginosa* (ATCC 27853), and *Klebsiella pneumoniae* (ATCC 43816) were tested. Additionally, the antibiotic-resistant strains, methicillin-resistant *S. aureus* (MRSA, NCTC 12493), *K. pneumoniae* (ATCC BAA 1705), and *K. pneumoniae* (ATCC BAA 2342), were also employed to investigate the potential application of all peptides against antibiotic resistance. All the bacterial strains were inoculated in Mueller Hinton broth (MHB), pH 7.4 (Oxiod, Basingstoke, UK) at 37 °C. Then, the log-phase bacteria were mixed with the peptide at the concentrations from 512 to 1 µM in two-fold dilution, in a 96 well-plate. The wells contained sterilized MHB and bacteria cultures were employed as a blank and growth control, respectively. Besides this, gentamycin, vancomycin, and norfloxacin were applied as positive controls.

Furthermore, the antimicrobial activity in the presence of MgCl_2_, CaCl_2_, NaCl and 10% fetal bovine serum (FBS) was investigated. In detail, 2 and 5 mM of MgCl_2_ and CaCl_2_, and 150 and 375 mM NaCl, were applied, respectively. The supplements were added into the medium that was used for diluting the bacteria suspension. Then, the diluted bacteria culture was mixed with the peptide stock solution in the 96-well plate, as mentioned above. The assay was performed three times and each assay contained 5 replicates.

### 4.5. Enzyme Stability Assay

The peptides were dissolved in phosphate-buffered saline (PBS) and mixed with the respective enzyme (trypsin and chymotrypsin) with a ratio of 100:1 (*m*/*m*). Then, 135 µl of the reaction solution was transferred into a new vial. The hydrolysis reaction was immediately terminated by adding 15 µl of 10% TFA at the different time points (0, 5, 10, 20, 40, 60, 120 min). The mixture was then analyzed by reverse-phase HPLC and respective peak areas were calculated to obtain the residual amount of peptides at different times.

### 4.6. Cytotoxicity Assay

The cytotoxicity assay was performed using Pierce™ LDH cytotoxicity assay kit (Thermo Fisher Scientific, UK). The MTT assay was performed with a typical method as described in the previous study [58]. Human keratinocyte cell line HaCat was treated with DMPC-10A and DMPC-10B at concentrations from 128 to 1 μM and 1% Triton X-100 and PBS were set as a positive and a negative control, respectively. For MTT assay, the formazan was dissolved by DMSO and detected at 540 nm using the plate reader. The release of LDH was determined by Synergy HT plate reader (490 nm) (Biolise BioTek EL808, Winooski, VT, USA) after a 6 h incubation at 37 °C. Moreover, 1% Triton X-100 and PBS were set as a positive and a negative control, respectively.

### 4.7. Hemolysis Test

The hemolytic activity of each peptide was measured by incubating a range of peptide concentrations from 512 to 1 µM in a two-fold dilution in a 2% suspension of the horse erythrocytes, as described in a previous study [59]. Briefly, 200 µL of peptide solution of each concentration was mixed with 200 µL of pre-washed erythrocyte suspension. Then, 5 µL of Triton X-100 was added to 195 µL of PBS as a positive control and then mixed with 200 µL of the erythrocyte suspension. The negative control was set as 200 µL of PBS mixed with 200 µL of the erythrocyte suspension. Then, 100 µl of the supernatant from each sample was transferred to a microtiter plate after 2 h incubation and the absorbance was measured by a Synergy HT plate reader at 550 nm (BioTek, Minneapolis, MN, USA).

### 4.8. Membrane Permeability Kinetic Assay

The permeability of the bacterial cell membrane was assessed by the uptake of a nucleic fluorescent dye, SYTOX^TM^ Green (Thermo Fisher Scientific, Waltham, MA, USA), which was described in a previous study [58]. The bacteria were cultured to log phase and harvested by centrifugation at 1000 rcf for 20 min. Followed by a wash step using 5% TSB/0.85% NaCl solution, the bacterial cells were resuspended using the same solution and diluted to a OD value of 0.8 at 590 nm. The prepared bacterial culture was subsequently mixed with the peptides and the dye in a black 96-well plate and then analyzed by excitation and emission wavelengths of 485 and 528 nm at 37 °C for 120 min (interval 5 min), using a Synergy HT plate reader (BioTek, Minneapolis, MN, USA).

Additionally, the dipalmitoylphosphatidylcholine (DPPC) small unilamellar vesicles (SUVs) were prepared as described previously [60]. Then, 10 mg/mL DPPC dissolved in 1:1 (*v*/*v*) methanol/chloroform was evaporated and hydrated in HEPES buffer to form the multilamellar vesicles (MLVs). Then, the small unilamellar vesicles (SUVs) were achieved by sonication. The prepared DPPC SUVs were added to the well to achieve a final concentration of 1 mg/mL.

The experiment contained 5 replicates. The 100% membrane permeabilization was achieved by treatment with 70% isopropanol. The bacteria suspension mixed with SYTOX dye only was employed as a negative control.

### 4.9. Antibiofilm Assays

The antibiofilm activity of synthetic peptides was evaluated via the inhibitory effect against the formation of biofilm and the eradication of mature biofilm, which were performed as the previously study [58] with minor modifications. With regard to the inhibition of biofilm formation, the peptides were mixed with diluted bacteria culture in a 96-well plate and incubated at 37 °C for 24–48 h. Then, the solution in each well was discarded. The biofilm was washed with PBS. Finally, the biofilm in each well was stained by 100 μL of 0.1% crystal violet solution and further dissolved by 30% acetic acid. The absorbance of dissolved crystal violet in each well was recorded at 595 nm. For the eradication of mature biofilm, biofilm was formed with incubation at 37 °C for 24–48 h. Then, the plate was washed with PBS and filled with the peptides which were prepared in the fresh medium. After treatment for 24 h at 37 °C, the plate was stained as mentioned above. Both assays were repeated three times, with 5 replicates each time.

### 4.10. Evaluation of Combination Effects of DMPC-10B

To evaluate the potential synergetic effect of DMPC-10B with the conventional antibiotics, gentamycin and norfloxacin, against *K. pneumoniae* (ATCC 43816), *K. pneumoniae* (ATCC BAA 1705), and *K. pneumoniae* (ATCC BAA 2342), a checkerboard assay was applied, as described previously [61,62]. The bacteria culture was prepared and diluted as performed in Section 4.4. Then, the bacteria dilution was mixed with a different combination of DMPC-10B and the antibiotics in the 96-well plate. The wells only contained individual peptides or antibiotics were applied as reference controls. Similarly, the turbidity of each well in the plate was measured at OD 550 nm, after incubation at 37 °C for 24 h. The fractional inhibitory concentration index (FICI) of each combination was defined as follows:

$$\mathrm{FICI}=\frac{\mathrm{MIC}\;\mathrm{of}\;\mathrm{DMPC}-10B\;\mathrm{in}\;\mathrm{combination}}{\mathrm{MIC}\;\mathrm{of}\;\mathrm{DMPC}-10B\;\mathrm{alone}}+\frac{\mathrm{MIC}\;\mathrm{of}\;\mathrm{antibiotic}\;\mathrm{in}\;\mathrm{combination}}{\mathrm{MIC}\;\mathrm{of}\;\mathrm{antibiotic}\;\mathrm{alone}}$$

The profile of the combination was interpreted as synergistic, additive, and antagonistic for FICI ≤ 0.5, 0.5 < FICI ≤ 4.0, and FICI > 4.0, respectively.

### 4.11. Assessing the Efficacy of DMPC-10B against MRSA and K. Pneumoniae Strains In Vivo

The assessment of in vivo antimicrobial activity of DMPC-10B was performed using the larva of *Galleria mellonella*, as in the previous study, with minor modifications [63]. The infection model was constructed by injecting 10 μL of MRSA (NCTC 12493) and *K. pneumoniae* (ATCC 43816) bacteria suspension (5 × 10^7^ CFU/mL) which was prepared in PBS. After 1 h, each infected larva was further administered an injection of 10 μL of peptide solution at different doses of 5, 10, 20 mg/kg. The infected larva that was administered 10 μL of PBS was employed as a negative control, while 20 mg/kg of gentamicin was used as a positive control. Each group contained 10 larvae and all larvae were inspected every 24 h for 5 days (the first day was 12 h).

## 5. Conclusions

As the major problem of antibiotic resistance worldwide has become serious, versatile therapeutics and approaches have emerged for the development of antimicrobials. Herein, we demonstrated a broad-spectrum and potent antimicrobial peptide, DMPC-10B, with negligible hemolytic activity and cytotoxicity. It revealed a potent effect against wildtype bacteria strains as well as the antibiotic-resistant bacteria in vitro. Additionally, it exhibited a high degree of resistance to hydrolysis by trypsin and chymotrypsin, which could overcome the drawback for oral administration of protein and peptide. Additionally, DMPC-10B exerted synergistic activity with gentamicin against *K. pneumoniae* KPC strains, which might benefit clinical therapy in the treatment of multidrug-resistant pathogens. Considering this remarkable feature, it is believed that DMPC-10B might be an optimized AMP candidate, compared with the other naturally occurring AMPs with obvious cytotoxicity and low bio-stability. Although, in this study, the larvae model may not reflect the real antimicrobial efficacy of DMPC-10B in vivo, these results bring insights into the potential of the development of new antibiotic alternatives.

## Figures and Tables

**Table 1:** The minimal inhibitory concentrations (MICs) and minimal bactericidal concentrations (MBCs) of DMPC-10A and DMPC-10B against selected microorganisms. MICs of antibiotics (gentamicin, vancomycin and norfloxacin) were tested as well.

Strains		MICs/MBCs (μM)		MICs (µg/mL)/(μM)		
		DMPC-10B	DMPC-10A	Gentamicin	Vancomycin	Norfloxacin
*Gram-positive bacteria*	*S. aureus* *(NCTC 10788)*	4/16	4/8	<0.0625/<0.13	0.125/0.08	2/6.26
	*MRSA* *(NCTC 12493)*	4/8	8/16	0.125/0.26	0.125/0.08	2/6.26
	*E. faecalis* *(NCTC 12697)*	64/64	64/64	4/8.38	1/0.69	4/12.53
*Gram-negative bacteria*	*E. coli* *(NCTC 10418)*	2/4	8/8	1/2.09	>32/>22.07	1/3.13
	*K. pneumoniae* *(ATCC 43816)*	8/8	4/64	1/2.09	>32/>22.07	2/6.26
	*K. pneumoniae* *(ATCC BAA 1705)*	32/32	32/32	2/4.19	>32/>22.07	>32/>100.21
	*K. pneumoniae* *(ATCC BAA 2342)*	16/16	16/16	4/8.38	>32/>22.07	>32/>100.21
	*P. aeruginosa* *(ATCC 27853)*	4/32	4/4	0.25/0.52	>32/>22.07	2/6.26

**Table 2:** Effect of MgCl_2_, CaCl_2_, NaCl and fetal bovine serum (FBS) on the antimicrobial activity of DMPC-10B against *S. aureus* (NCTC 10788) and *E. coli* (NCTC 10418).

Additive	Concentration	MICs of DMPC-10B (µM)		MICs of DMPC-10A (µM)	
		*S. aureus*	*E. coli*	*S. aureus*	*E. coli*
None	-	4	2	4	8
MgCl_2_	2 mM	32	16	32	32
	5 mM	64	32	64	64
CaCl_2_	2 mM	32	16	32	32
	5 mM	64	32	64	64
NaCl	150 mM	64	16	32	16
	375 mM	128	32	64	32
FBS	10%	8	4	16	16

**Table 3:** Synergistic effect of DMPC-10B with gentamicin and norfloxacin against the growth of *K. pneumoniae* (ATCC 43816), *K. pneumoniae* (ATCC BAA 1705), and *K. pneumoniae* (ATCC BAA 2342). The MICs are shown as the combined MIC/individual MIC. The fractional inhibitory concentration index (FICI) was calculated and interpreted as synergistic for FICI ≤ 0.5, additive for 0.5 < FICI ≤ 4.0, and antagonistic for FICI > 4.0.

Combination	Bacteria Strains		
	*K. pneumoniae* (ATCC 43816)	*K. pneumoniae* (ATCC BAA 1705)	*K. pneumoniae* (ATCC BAA 2342)
DMPC-10B	1/8	4/32	2/16
Gentamicin	0.25/1	0.5/2	1/4
FICI (DMPC-10B/Gentamicin)	0.375	0.375	0.375
DMPC-10B	4/8	32/32	16/16
Norfloxacin	0.125/1	0.0625/>32	0.0625/>2
FICI (DMPC-10B/Norfloxacin)	0.625	>1	>1

## Supplementary Materials

The following are available online at https://www.mdpi.com/2079-6382/9/9/627/s1; Figure S1. The cell viability of tested microorganisms in the treatment of DMPC-10A and DMPC-10B at concentrations from 512 to 1 µM. The error bar represents the standard deviation (SD) of 15 replicates in three tests (5 replicates for each). The cell viability of bacteria treated with medium only was regarded as 100% viability; Figure S2. The cell viability of *S. aureus* (NCTC 10788) and *E. coli* (NCTC 10418) in the treatment of DMPC-10A and DMPC-10B at concentrations from 512 to 1 µM with the presence of different concentrations of cations, including MgCl2, CaCl2, NaCl, and 10% FBS. The error bar represents the standard deviation (SD) of 15 replicates in three tests (5 replicates for each). The cell viability of bacteria treated with medium only was regarded as 100% viability; Figure S3. Reverse-phase HPLC chromatograms of 100 μL 1mg/mL peptide solution treated with 1% (*m*/*m*) trypsin and chymotrypsin within 120 min. Ten percent of 10% trifluoroacetic acid was used to stop the reaction between enzyme and peptides. 0 min was the sample which only contained peptide solution and 10% trifluoroacetic acid; Figure S4. Molecular docking analysis of the interaction of DMPC-10A (A and C) and DMPC-10B (B and D) with POPC (A and B) and POPE (C and D) lipid bilayer. The calculated binding affinity is −3.4 kcal/mol (A), −3.4 kcal/mol (B), −3.9 kcal/mol (C), and −4.8 kcal/mol (D), respectively; Figure S5. The turbidity of culture at OD 550nm in the treatment of the combination of DMPC-10B and gentamicin (a–c) or norfloxacin (d–f) against *K. pneumoniae* (ATCC 43816) (a,d), *K. pneumoniae* (ATCC BAA 1705) (b,e), and *K. pneumoniae* (ATCC BAA 2342) (c,f). The error bar represents the standard deviation (SD) of 5 replicates.
