# Supplementary material for: Broad-Spectrum Antimicrobial Activity and Improved Stability of a D-Amino Acid Enantiomer of DMPC-10A, the Designed Derivative of Dermaseptin Truncates
Source: Antibiotics (Basel). 2020 Sep 21;9(9):627. doi: 10.3390/antibiotics9090627 (PMC7557582; doi:10.3390/antibiotics9090627)
Supplement: Supplementary file 1 [file antibiotics-09-00627-s001.pdf]

Broad-spectrum antimicrobial activity and improved stability of a D-amino acid enantiomer of DMPC-10A, the designed derivative of dermaseptin truncates

Supplementary Materials

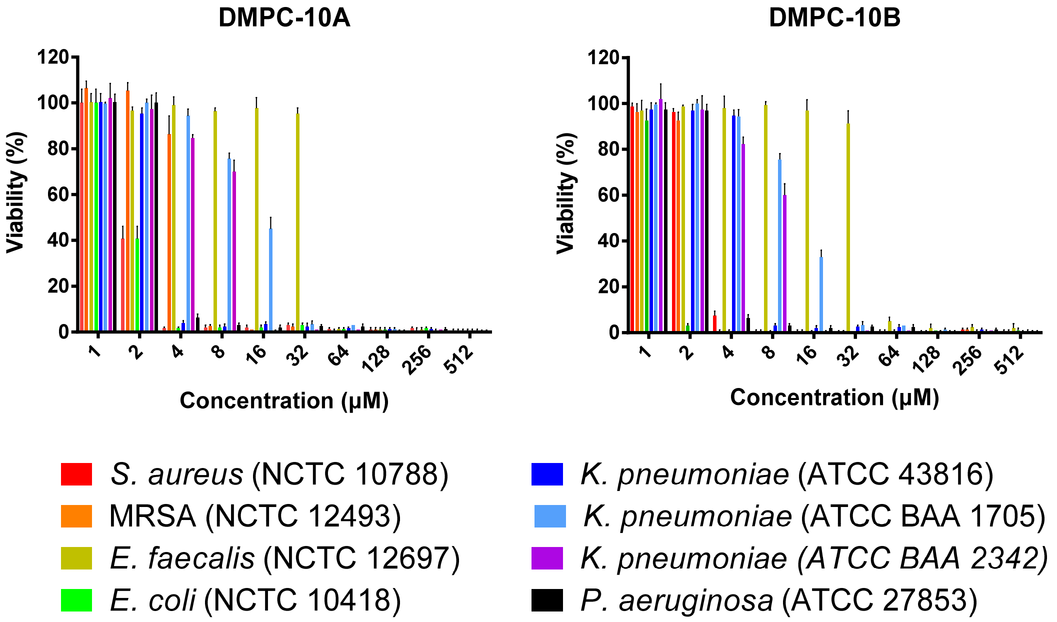

Figure S1. The cell viability of tested microorganism in the treatment of DMPC-10A and DMPC-10B at the concentration from 512 to 1 μM. The error bar represents the standard deviation (SD) of 15 replicates in three tests (5 replicates for each).

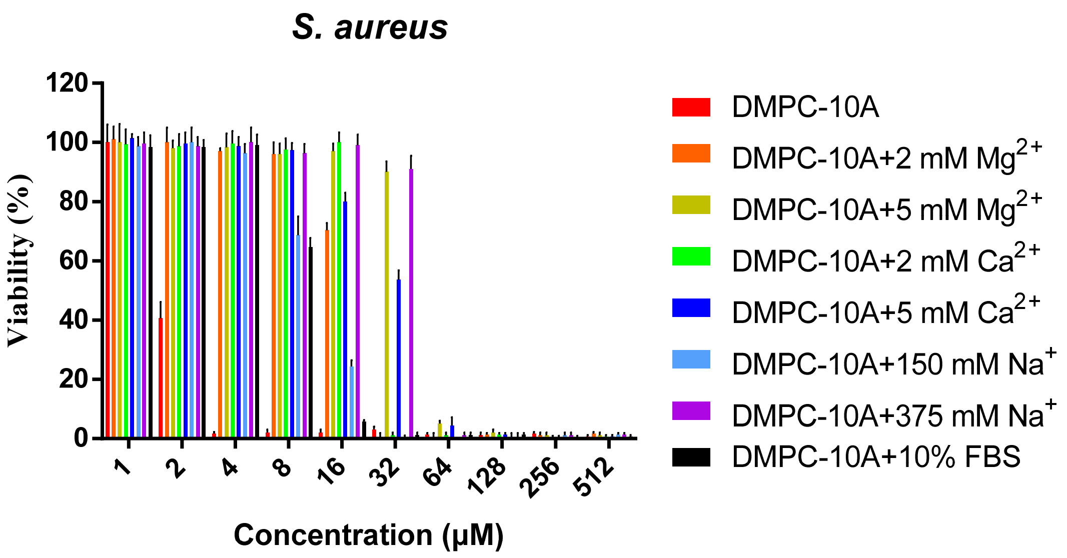
S. aureus
120
100
80
60
40
20
0
Viability (%)
1
2
4
8
16
32
64
128
256
512
Concentration (μM)
DMPC-10A
DMPC-10A+2 mM Mg2+
DMPC-10A+5 mM Mg2+
DMPC-10A+2 mM Ca2+
DMPC-10A+5 mM Ca2+
DMPC-10A+150 mM Na+
DMPC-10A+375 mM Na+
DMPC-10A+10% FBS

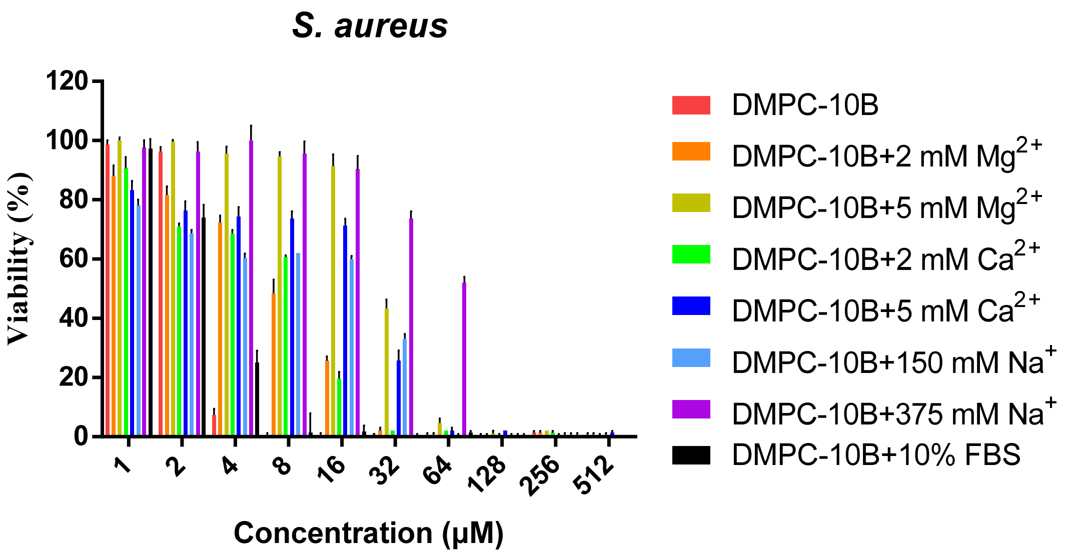
S. aureus
120
100
80
60
40
20
0
Viability (%)
1
2
4
8
16
32
64
128
256
512
Concentration (μM)
DMPC-10B
DMPC-10B+2 mM Mg2+
DMPC-10B+5 mM Mg2+
DMPC-10B+2 mM Ca2+
DMPC-10B+5 mM Ca2+
DMPC-10B+150 mM Na+
DMPC-10B+375 mM Na+
DMPC-10B+10% FBS

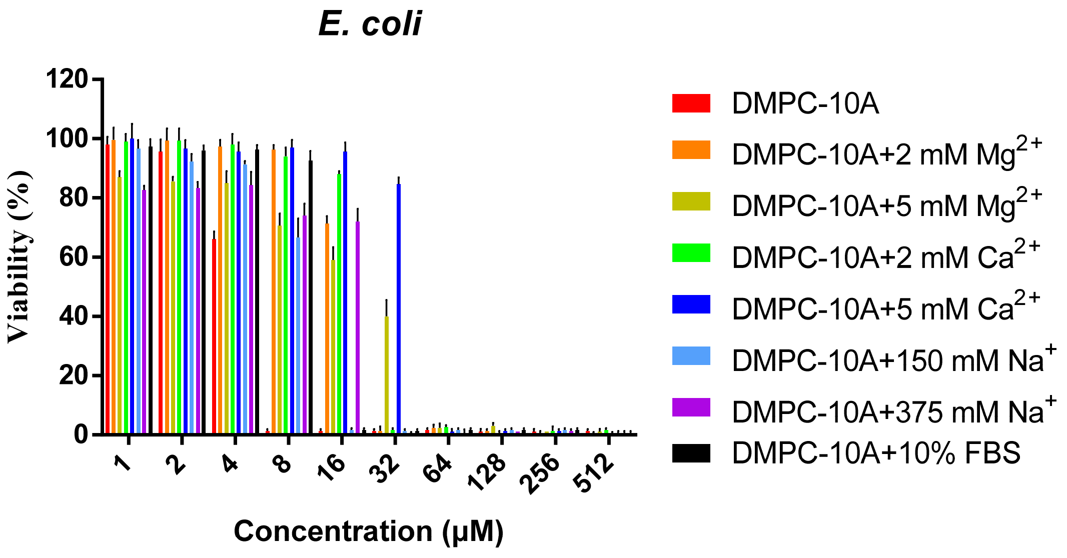
E. coli
120
100
80
60
40
20
0
Viability (%)
1
2
4
8
16
32
64
128
256
512
Concentration (μM)
DMPC-10A
DMPC-10A+2 mM Mg2+
DMPC-10A+5 mM Mg2+
DMPC-10A+2 mM Ca2+
DMPC-10A+5 mM Ca2+
DMPC-10A+150 mM Na+
DMPC-10A+375 mM Na+
DMPC-10A+10% FBS

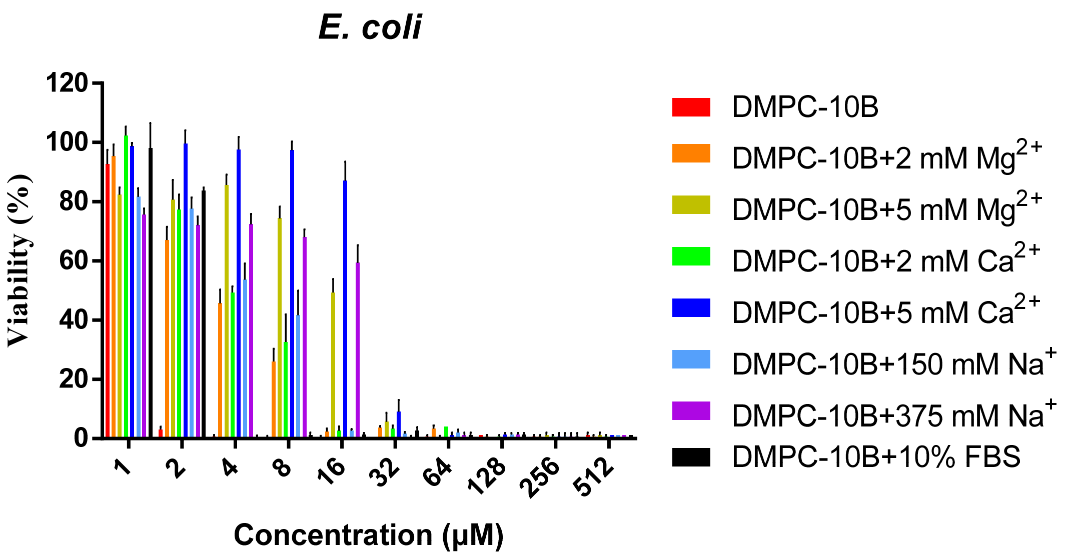

Figure S2. The cell viability of *S. aureus* (NCTC 10788) and *E. coli* (NCTC 10418) in the treatment of DMPC-10A and DMPC-10B at the concentration from 512 to 1 μM with the presence of the different concentrations of the cations, including MgCl2, CaCl2, NaCl, and 10% FBS. The error bar represents the standard deviation (SD) of 15 replicates in three tests (5 replicates for each).

Trypsin+DMPC-10A

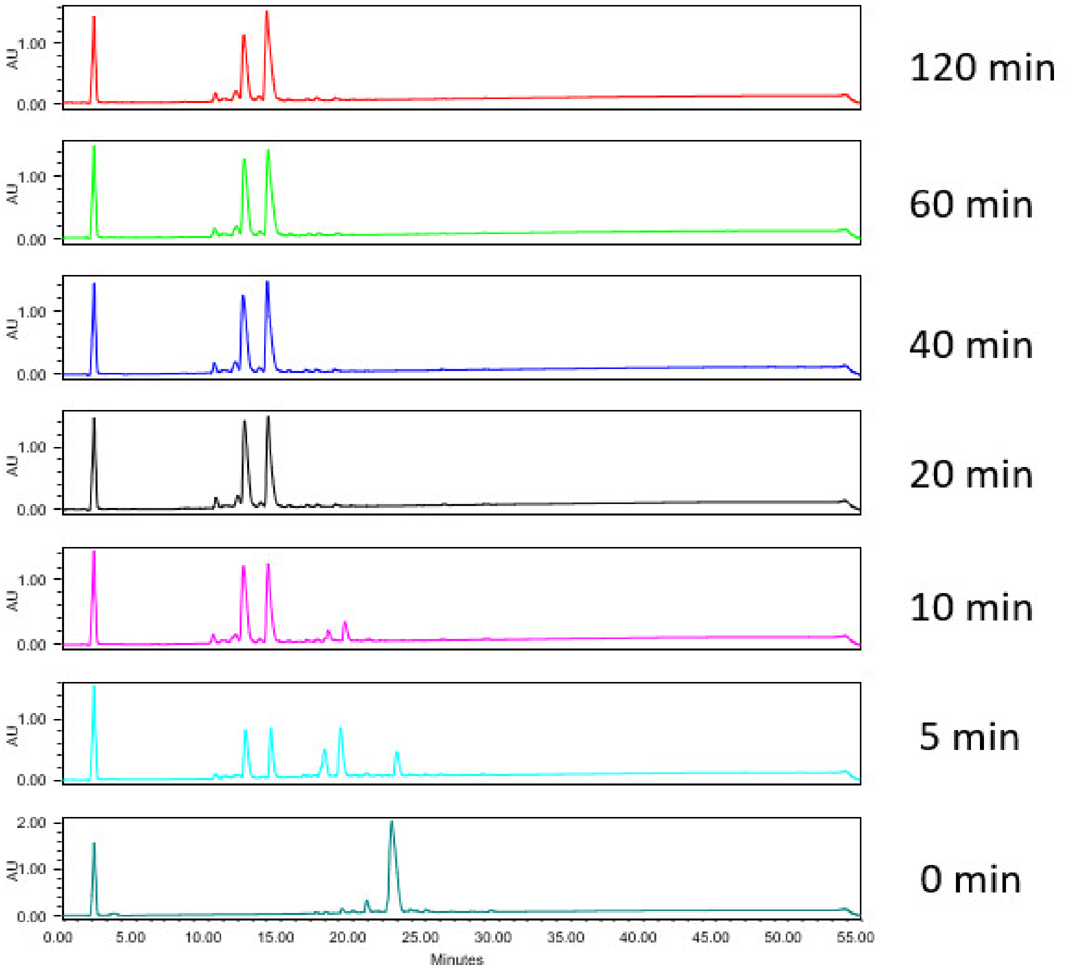

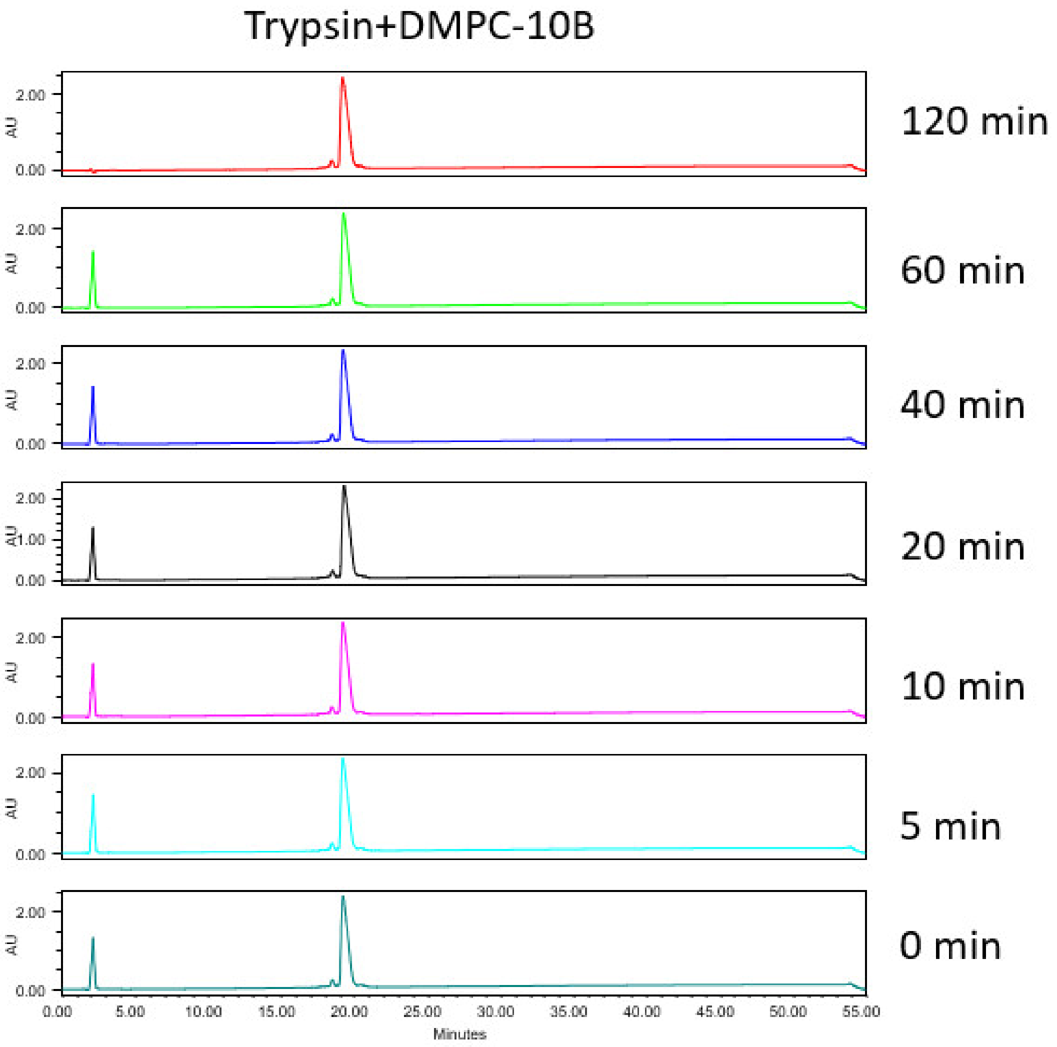
Trypsin+DMPC-10B
120 min
60 min
40 min
20 min
10 min
5 min
0 min
AU
2.00
1.00
0.00
Minutes
0.00
5.00
10.00
15.00
20.00
25.00
30.00
35.00
40.00
45.00
50.00
55.00

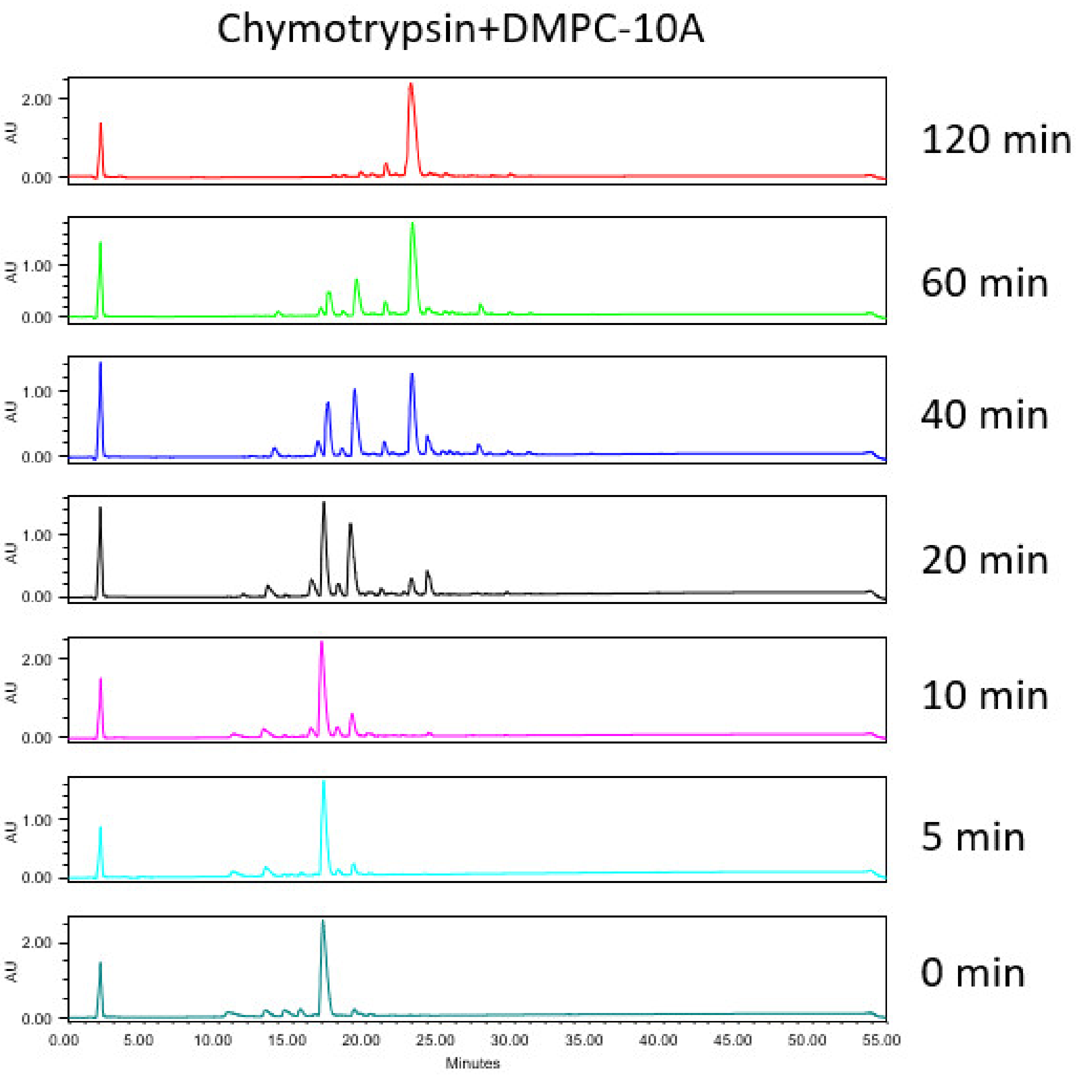
Chymotrypsin+DMPC-10A
120 min
60 min
40 min
20 min
10 min
5 min
0 min
AU
2.00
1.00
0.00
0.00
5.00
10.00
15.00
20.00
25.00
30.00
35.00
40.00
45.00
50.00
55.00
Minutes

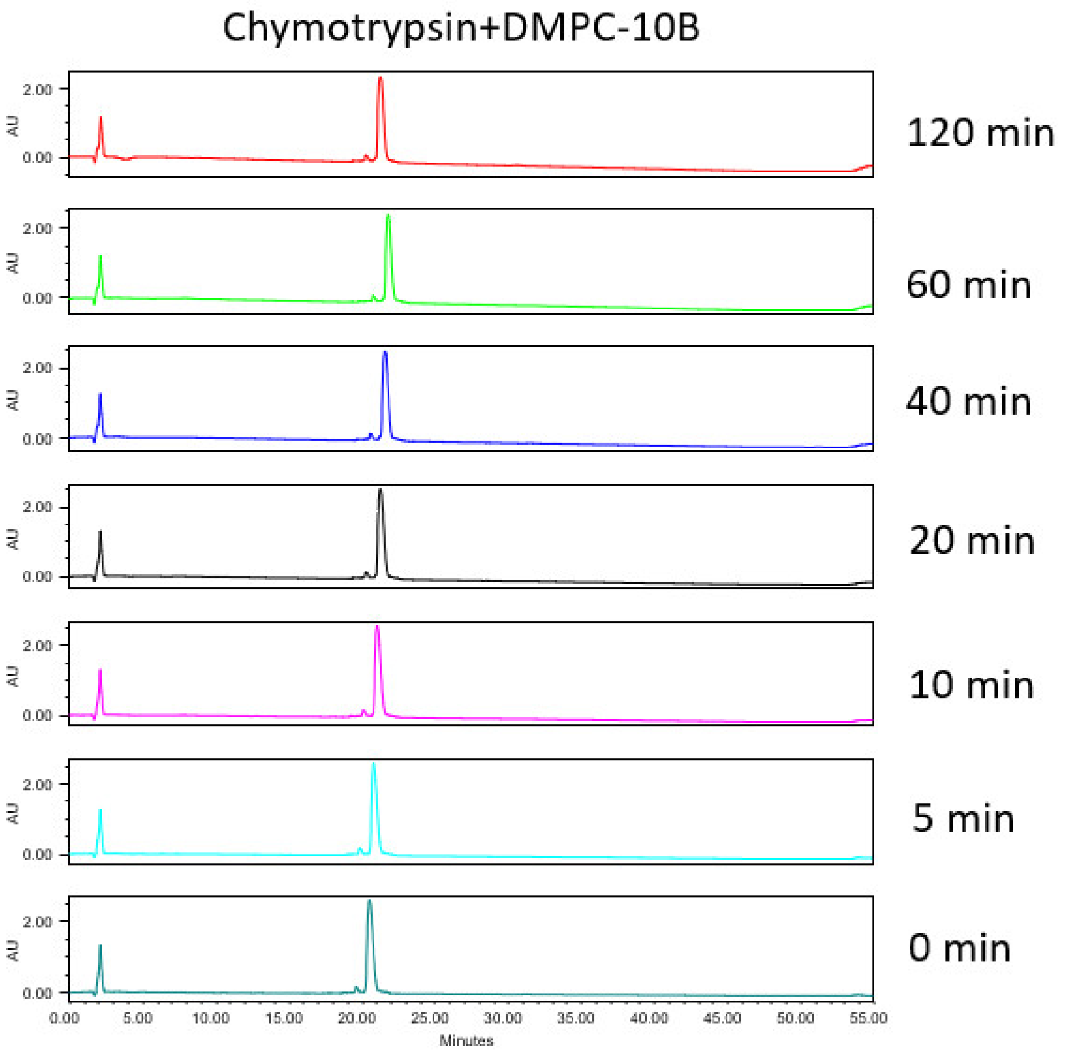

Figure S3. Reverse phase HPLC chromatograms of 100 μl 1mg/mL peptide solution treated with 1% (m/m) trypsin and chymotrypsin within 120 minutes. Ten percent of 10% trifluoroacetic acid was used to stop the reaction between enzyme and peptides. 0 min was the sample only contained peptide solution and 10% trifluoroacetic acid.

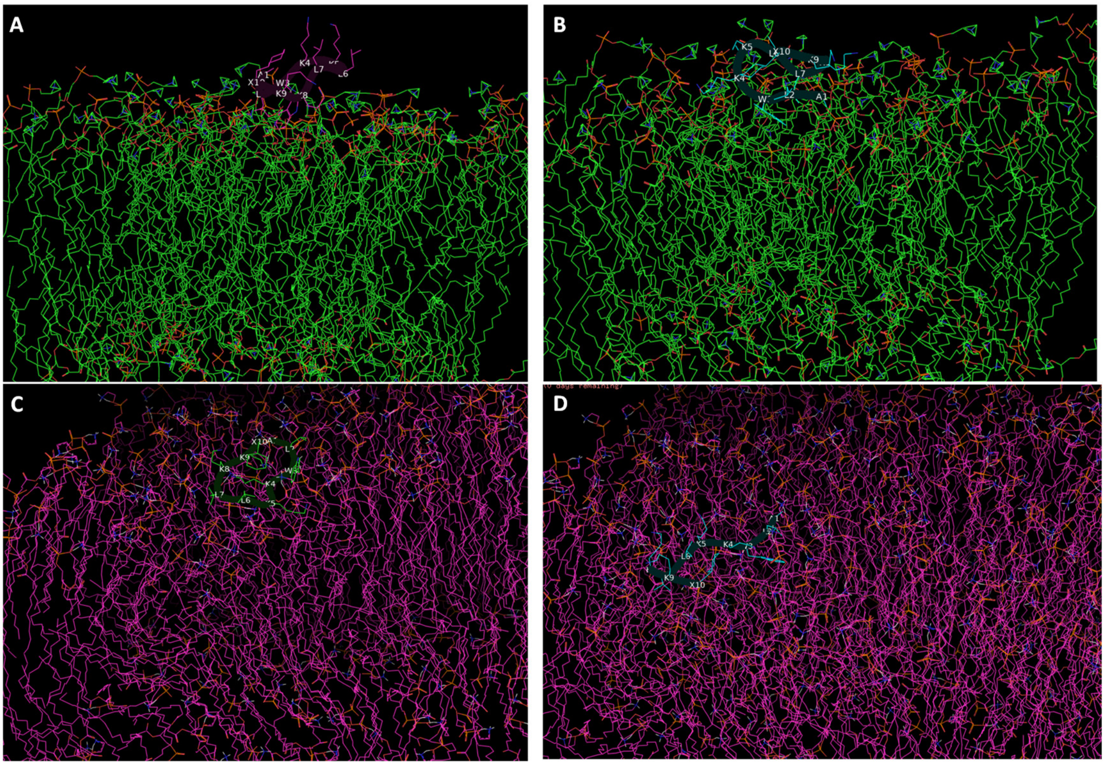

Figure S4. Molecular docking analysis of the interaction of DMPC-10A (A and C) and DMPC-10B (B and D) with POPC (A and B) and POPE (C and D) lipid bilayer. The calculated binding affinity is -3.4 kcal/mol (A), -3.4 kcal/mol (B), -3.9 kcal/mol (C) and -4.8 kcal/mol (D), respectively.

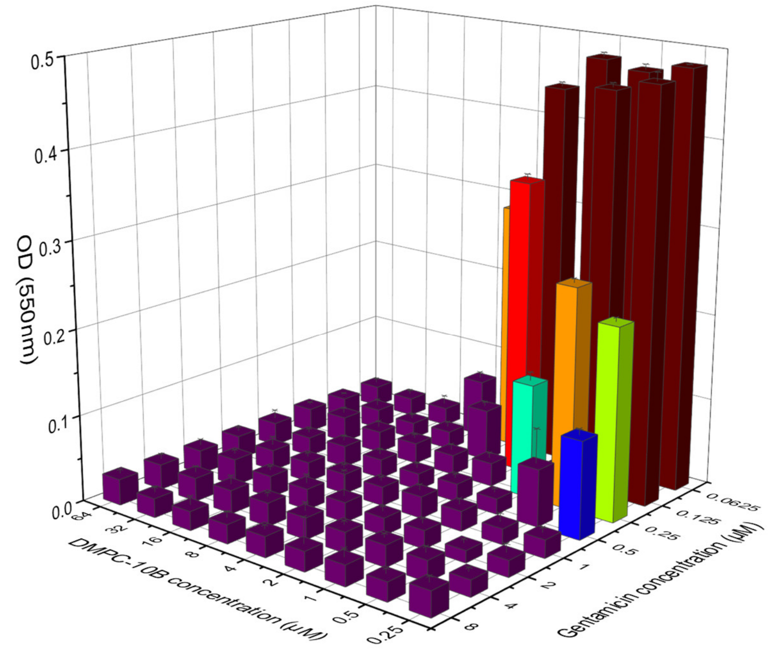

OD (550nm)
0.0
0.1
0.2
0.3
0.4
0.5
DMPC-10B concentration (μM)
64
32
16
8
4
2
1
0.5
0.25
Gentamicin concentration (μM)
0.0625
0.125
0.25
0.5
1
2
4
8

(a)

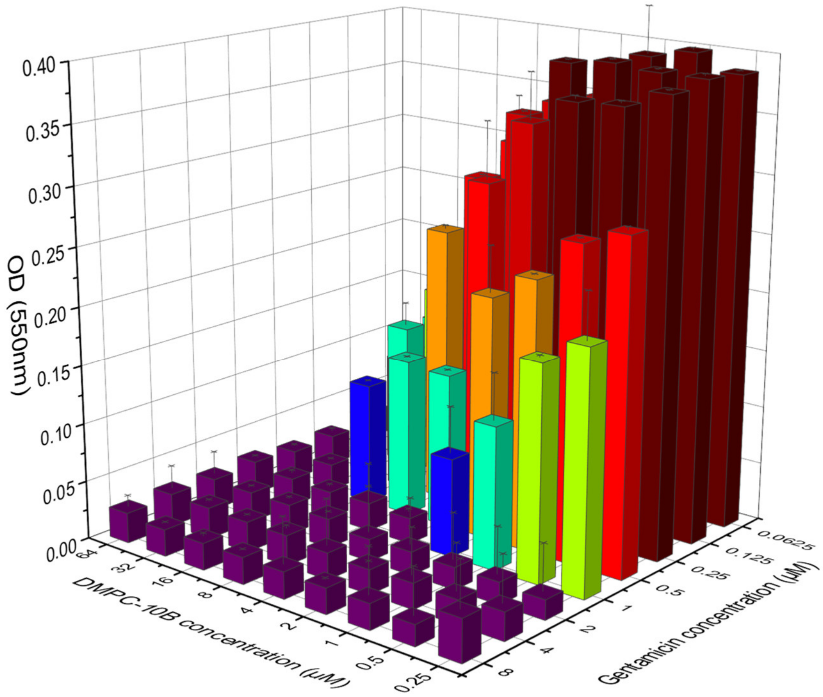

OD (550nm)
0.00
0.05
0.10
0.15
0.20
0.25
0.30
0.35
0.40
DMPC-10B concentration (μM)
64
32
16
8
4
2
1
0.5
0.25
Gentamicin concentration (μM)
0.0625
0.125
0.25
0.5
1
2
4
8

(b)

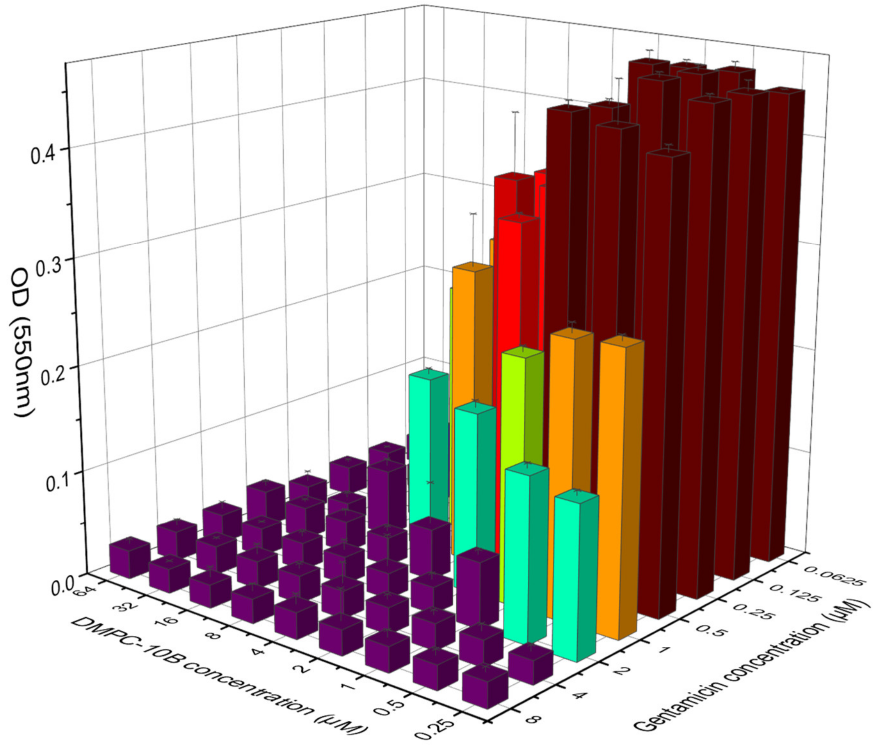
OD (550nm)
0.0
0.1
0.2
0.3
0.4
DMPC-10B concentration (μM)
64
32
16
8
4
2
1
0.5
0.25
Gentamicin concentration (μM)
8
4
2
1
0.5
0.25
0.125
0.0625

(c)

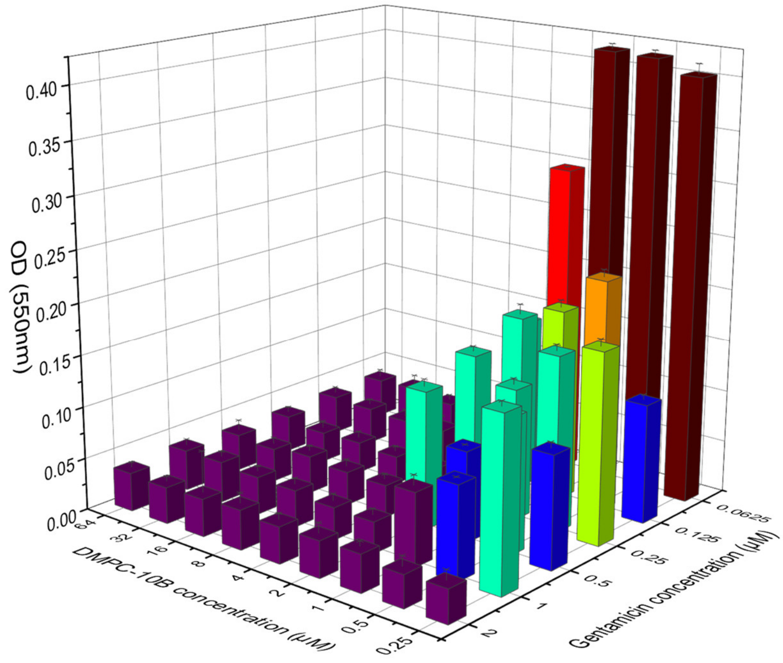
OD (550nm)
0.00
0.05
0.10
0.15
0.20
0.25
0.30
0.35
0.40
DMPC-10B concentration (μM)
64
32
16
8
4
2
1
0.5
0.25
Gentamicin concentration (μM)
2
1
0.5
0.25
0.125
0.0625

(d)

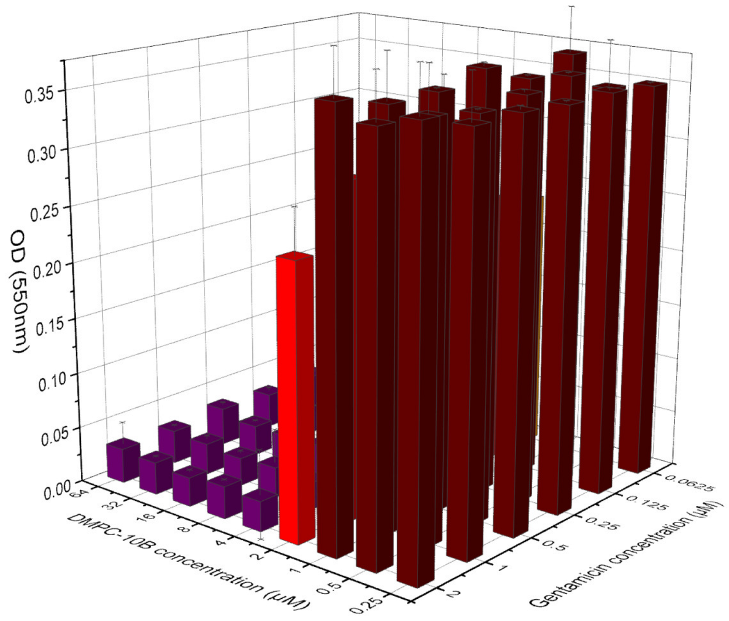

(e)

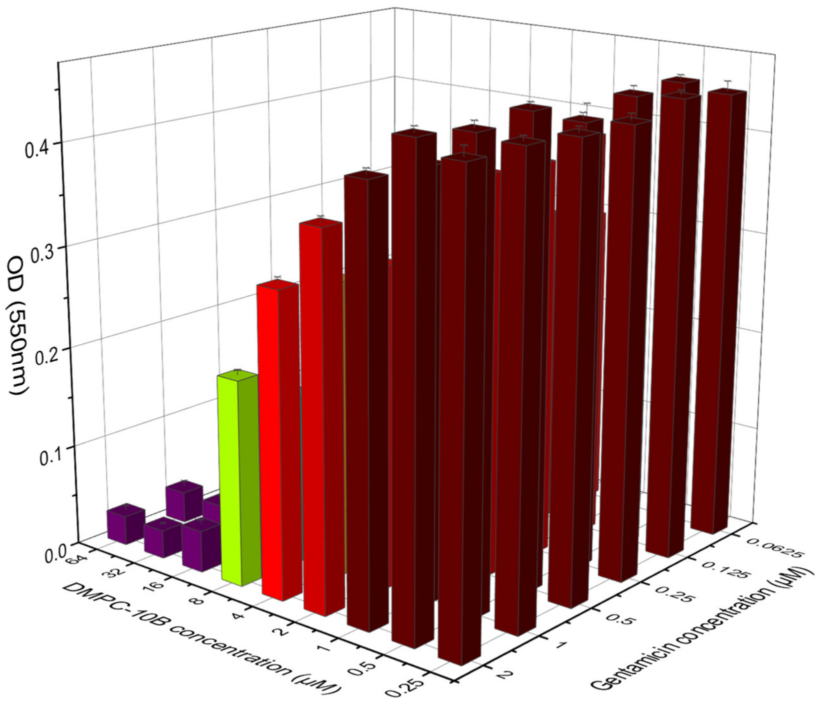

(f)

Figure S5. The turbidity of culture at OD 550nm in the treatment of the

combination of DMPC-10B and gentamicin (a, b, and c), or norfloxacin (d, e, and f) against *K. pneumoniae* (ATCC 43816) (a & d), *K. pneumoniae* (ATCC BAA 1705) (b & e) and *K. pneumoniae* (ATCC BAA 2342) (c & f). The error bar represents the standard deviation (SD) of 5 replicates.
